# Decapitation Rapidly Triggers Axillary Bud Release via Regulatory Network Reprogramming in *Nicotiana tabacum*

**DOI:** 10.3390/plants14243830

**Published:** 2025-12-16

**Authors:** Bingxin Xu, Qingsong Liu, Genhong Wang, Siyu Shao, Ping Zhao, Qingyou Xia

**Affiliations:** Integrative Science Center of Germplasm Creation in Western China (CHONGQING) Science City, Chongqing Technology Innovation Center of Breeding, Biological Science Research Center, Southwest University, Chongqing 400715, China; xubingxin5163@163.com (B.X.); liuqingsong@swu.edu.cn (Q.L.); wanggh168@swu.edu.cn (G.W.); shaosiyu711@163.com (S.S.)

**Keywords:** axillary bud, transcriptome, dormancy release, dynamic regulatory network

## Abstract

Axillary buds are key organs that determine shoot branching and aerial architecture in plants and critically influence crop growth and productivity. Understanding the molecular mechanisms underlying the transition from dormancy to bud activation is a central question in plant developmental regulation. Although previous studies have revealed post-release developmental processes, the early regulatory network that triggers dormancy release remains unclear. In this study, we used tobacco (*Nicotiana tabacum*) as a model and focused on transcriptomic changes of regulatory factors in axillary buds within 36 h after decapitation. Then, we systematically analyzed key molecular events that induce dormancy release. The results revealed the involvement of diverse signals in decapitation-induced bud activation, including key plant hormones like auxin, cytokinin, and gibberellin; as well as external cues such as sugar, nitrogen, and light. Significant changes occurred as early as 0.5 to 1 h after decapitation. Among these, auxin and sugar signaling played central roles in initiating dormancy release. In addition, various signaling factors exhibited coordinated regulatory effects during the continued development of activated buds. Functional validation further demonstrated that *EXB1* and *STM*, two key regulators of axillary bud initiation, participated in the subsequent stages of branch development. In conclusion, our study reveals that decapitation-induced dormancy release of axillary buds occurs at a very early stage (0.5–1 h). This rapid response is driven by a complex regulatory network involving multiple hormones and metabolic signals. These findings provide new molecular insights into the dynamic regulatory balance of axillary bud development. They also establish a theoretical basis and strategic reference for trait regulation and modular breeding design.

## 1. Introduction

Branching is one of the most important components of the plant shoot system. It determines plant structural traits, morphological diversity, environmental adaptability, and reproductive potential. In crops, branching is a key factor that affects yield per unit area. It is considered a major agronomic trait [1,2] and an important target for yield improvement in modern agriculture [3,4]. In higher plants, branches originate from axillary buds, whose development is regulated by a combination of internal signals (such as plant hormones and sugars) and external cues (such as light and nutrients) [5,6,7,8,9].

Under apical dominance, axillary buds usually remain dormant and must go through three stages—dormancy, dormancy release, and growth—before forming branches [10]. Apical dominance refers to the physiological phenomenon in which the main shoot tip preferentially uses resources and suppresses the growth of axillary buds [11]. When the shoot apex is removed, apical dominance is disrupted. Resources are then redistributed to axillary buds, promoting dormancy release and further development [12,13].

Auxin (IAA) is widely recognized as the central signal in maintaining apical dominance [14,15,16,17]. Two main models have been proposed to explain how auxin suppresses bud outgrowth: the canalization model and the second messenger model. According to the canalization model, auxin synthesized in the shoot apex’s young leaves is transported basipetally via the polar auxin transport stream (PATS). This process thereby establishes a strong auxin flow within the stem [18]. This leads to local auxin accumulation at the axil, which inhibits axillary bud outgrowth along the main axis [19,20]. When decapitation blocks this transport, auxin levels in the stem rapidly decline, and auxin efflux from buds increases. As a result, auxin content in the buds drops sharply, promoting dormancy release [19,21].

In the second messenger model, auxin from the shoot apex does not directly enter the axillary bud. Instead, it indirectly regulates bud activity through second messengers such as cytokinin (CK), strigolactone (SL), and sugars [5,16]. Auxin upregulates the expression of carotenoid cleavage dioxygenase genes *MAX3* and *MAX4*, thereby promoting SL biosynthesis and inhibiting bud growth [22,23,24]. SL can also block auxin efflux mediated by PIN proteins [25,26,27]. In addition, auxin represses CK biosynthesis by downregulating genes such as isopentenyl transferase (*IPT*) and cytokinin oxidase 2 (*CKX2*) [28,29]. CK and SL often act antagonistically in regulating axillary bud development [30,31,32], and this balance is mainly integrated by the transcription factor BRANCHED1 (BRC1) [33,34,35]. Studies in pea, rice, and Arabidopsis have shown that CK activates bud growth by repressing *BRC1* expression [33,35,36]. Concurrently, CK promotes auxin efflux by enhancing PIN protein accumulation and polarization, thus promoting dormancy release [37]. Gibberellin (GA) and abscisic acid (ABA) exert opposite roles in bud development. IAA positively regulates GA levels [38,39,40], while GA primarily promotes sustained bud growth after dormancy release [41]. ABA acts downstream of BRC1, is transported from upper leaves to lower buds, and inhibits bud growth [42,43]. It also suppresses bud outgrowth by inhibiting cell proliferation and auxin biosynthesis [44]. Sugars, especially sucrose, not only serve as energy sources but also act as key signals in bud dormancy regulation [45,46,47]. After decapitation, sugars rapidly redistribute along the stem and accumulate in axillary buds, a pattern that coincides with the timing of bud release [48]. Sugars can also influence hormone homeostasis by promoting CK biosynthesis and downregulating SL signaling genes such as *MAX2*, thereby facilitating dormancy release. Moreover, sucrose suppresses *BRC1* expression in a dose-dependent manner [49], further supporting its central role in regulating bud activation.

In addition to hormonal and sugar signals, environmental factors strongly influence branching. Nutrient elements such as nitrogen, phosphorus, and potassium, as well as light, affect axillary bud development. High nitrogen levels promote branching, depending on IAA, SL, and CK biosynthesis pathways [35,50,51]. High phosphorus levels inhibit SL biosynthesis [52,53]. Potassium transporters such as OsHAK5 regulate tillering in rice [54]. Low red to far-red light ratio (R:FR), commonly observed under dense planting conditions, suppresses axillary bud outgrowth and reduces branching [55,56]. Photoreceptors like FHY3 and FAR1 can relieve this inhibition by downregulating *BRC1* [57].

In summary, plant hormones, sugar signaling, and environmental cues interact to form a complex regulatory network. This integrated network precisely controls apical dominance and axillary bud development. Several hypotheses have been proposed: auxin as the dominant signal in apical dominance [17]; sugar as the initial trigger [48]; and the synergistic action of cytokinin and sugar, initiating dormancy release [58]. However, it remains unclear which signal first initiates bud dormancy release after apical dominance is removed. It is worth noting that most of these findings are derived from diploid model species such as Arabidopsis and pea. They are characterized by short life cycles and high genetic tractability. Thereby, they may not fully represent the branching mechanisms in polyploid or perennial species. And studies on perennial plants have focused on axillary bud growth at different developmental stages [59,60,61,62] or under prolonged stress treatments [63,64]. This work often involves omics approaches to investigate the underlying developmental patterns of the bud. However, the early regulatory events controlling dormancy release in buds remain insufficiently explored.

Decapitation is commonly employed in these studies to eliminate apical dominance. As a key agronomic practice, it is widely used as an agronomic practice to modify plant architecture and improve yield and quality in crops such as cotton (*Gossypium* spp.) [65], pepper (*Capsicum annuum* L.) [66], okra (*Abelmoschus esculentus*) [67], and tobacco (*Nicotiana tabacum*) [68]. Among these, tobacco, an allotetraploid species, exhibits strong apical dominance. It is commonly subjected to agronomic practices such as topping and bud removal. While previous studies in tobacco have mainly focused on the sustained growth of activated buds. For instance, Weifeng Wang et al. examined transcriptomic changes in axillary buds at 1, 3, and 5 days post-topping to elucidate the mechanisms underlying tobacco axillary bud development. Little is known about the early signals that trigger dormancy release [69,70,71].

In this study, we used tobacco as a model and performed RNA-seq analysis of axillary buds from 0.5 to 36 h after decapitation, focusing on the immediate early phase. We constructed a dynamic regulatory network for the early phase (0.5–1 h) of decapitation-induced bud dormancy release. In addition, we used CRISPR/Cas9 to validate the functions of key developmental genes, including *EXB1* and *STM*. Our results confirmed that bud release occurred at a very early stage after decapitation and was regulated by a complex interaction of multiple hormonal and metabolic signals. This study reveals the timing and signal logic of axillary bud dormancy release. It provides a new molecular framework for understanding the dynamic regulation of plant branching. This framework offers a theoretical basis for optimizing plant architecture and molecular breeding.

## 2. Results

### 2.1. Dynamic Changes in DEGs During Axillary Bud Development After Decapitation

**Figure 1:**
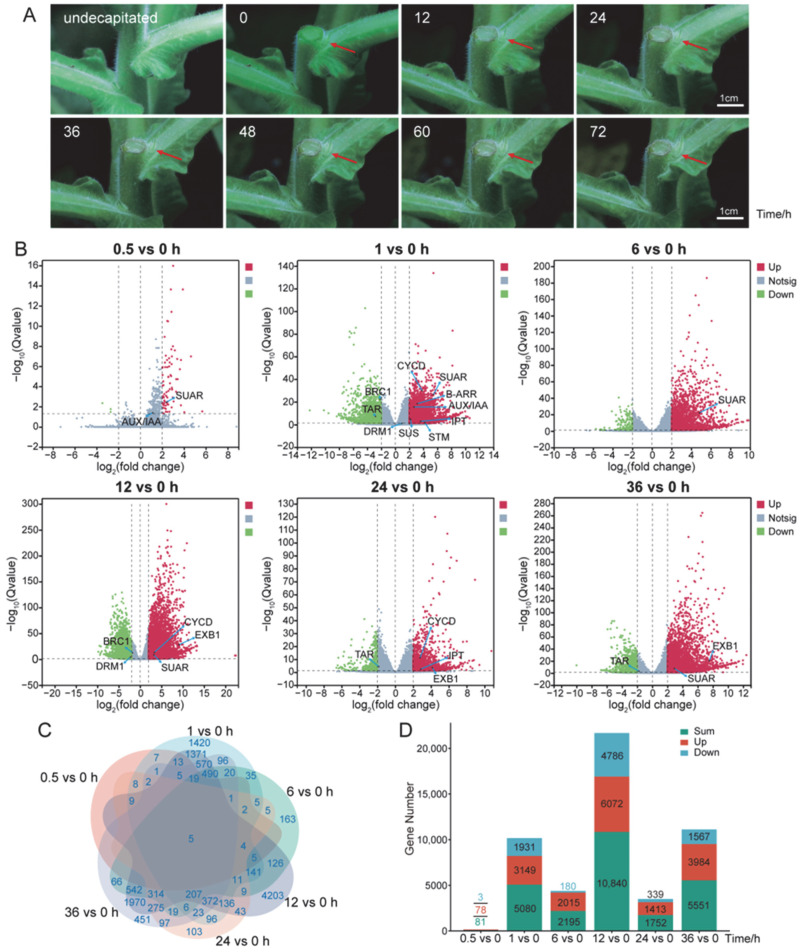
Transcriptome profiling of axillary buds in response to decapitation. (**A**) Phenotypic development of axillary buds in 12-leaf-stage tobacco plants (*Nicotiana tabacum* cv. Honghuadajinyuan) under untreated conditions and at different time points (0, 12, 24, 36, 48, 60, 72 h) after decapitation. Red arrows indicate axillary buds. Scale bar, 1 cm. (**B**) Volcano plots of differentially expressed genes (DEGs) in RNA-seq libraries from axillary buds of early-flowering-stage plants, comparing each post-decapitation time point to the 0 h control. Red and green dots represent significantly up- and down-regulated genes, respectively. Genes of interest (marked with blue arrows) are associated with: auxin (*TAR*, *AUX/IAA*, *SUAR*), cytokinin (*IPT*, *B-ARR*), and strigolactone (*BRC1*) pathways; sugar metabolism (*SUS*); and the regulation of cell cycle (*CYCD*), dormancy (*DRM1*), and axillary bud development (*STM*, *EXB1*). (**C**) Venn diagram illustrating the relationships among total DEGs across time points. (**D**) Bar chart showing the number of total DEGs. Red, green, and blue bars represent the counts of up-regulated, down-regulated, and total (up- and down-regulated) genes, respectively. Time unit: hours (h). DEGs were identified with thresholds of |log_2_ (fold change)| ≥ 2 and Q value < 0.05.

To investigate the dynamic process from dormancy release to branch development under decapitation, we conducted decapitation at the 12-leaf stage of tobacco (*Nicotiana tabacum* cv. Honghuadajinyuan). And we observed morphological changes in axillary buds at multiple time points (Figure 1A). Within 24 h of decapitation, the first and second node axillary buds exhibited marked swelling and elongation. By 36 h, their development had significantly accelerated. It suggests that axillary bud dormancy release is completed within 36 h post-decapitation, after which branch development initiates.

These results suggest that dormancy release in tobacco axillary buds is completed within 36 h after decapitation, followed by the onset of branch development. To uncover the early molecular responses of axillary buds triggered by decapitation, we collected bud samples of initial flowering stage at 0, 0.5, 1, 6, 12, 24, and 36 h post-decapitation for transcriptome sequencing. The RNA-seq data were of high quality, yielding an average of 1.19 Gb clean bases per sample with a Q30 score above 90%. The average mapping rates were 92.9% to the genome and 77.72% to genes (Table S1A1), confirming comprehensive and reliable sequencing coverage.

Differential expression analysis revealed substantial transcriptional changes at all time points. The number of differentially expressed genes (DEGs) ranged from 81 to 10,840, with most genes being upregulated (Figure 1B–D; Table S1A2). At 0.5 h post-decapitation, 81 DEGs were detected, indicating that the induction response occurred at a very early stage. At 12 h, the number of DEGs peaked at 10,840. Subsequently, 1752 and 5551 DEGs were identified at 24 and 36 h, respectively (Table S1A2), suggesting a sustained transcriptional reprogramming process. Volcano plot analysis showed time point-specific expression changes in multiple key genes after decapitation. These genes are associated with hormone and sugar metabolic pathways, as well as processes of cell cycle, dormancy, and axillary bud development (Figure 1B). Meanwhile, Venn diagram analysis showed that only five DEGs were shared across all time points (Figure 1C). It implies that most genes function in a time-specific manner rather than being persistently expressed.

These results indicate that decapitation rapidly triggers a strong transcriptional response in axillary buds, with the 0.5–12 h period representing the critical window for dormancy release. Bud development entered the branching phase within 36 h post-decapitation. Overall, axillary buds undergo extensive molecular remodeling during the early phase after decapitation. It provides a foundation for further exploration of the signaling pathways and key regulators involved in dormancy release.

### 2.2. Functional Enrichment Analysis of DEGs

**Figure 2:**
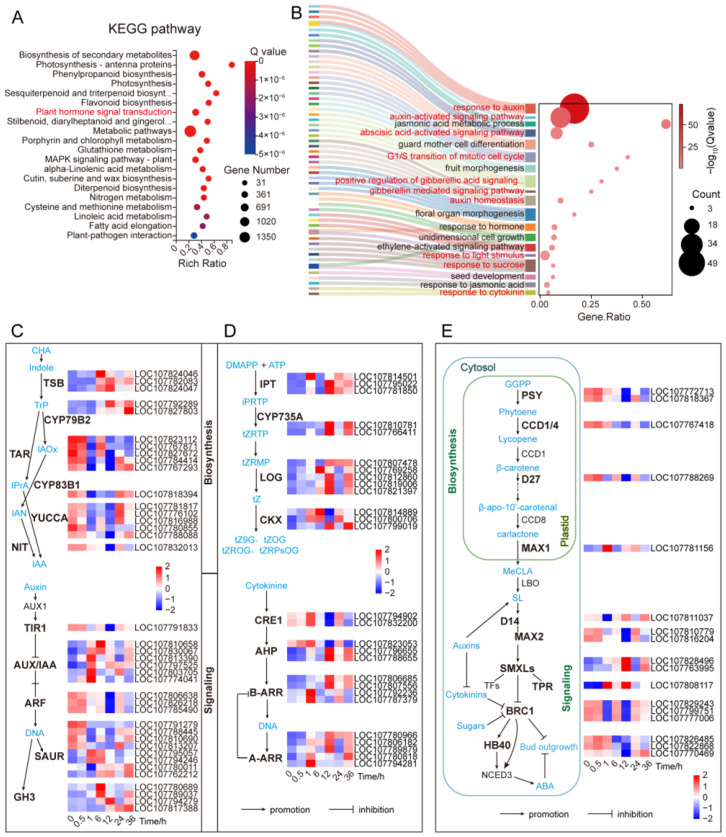
Analysis of differentially expressed genes (DEGs) across post-decapitation time courses. (**A**) KEGG enrichment analysis of total DEGs. Red and blue bubbles indicate significantly and non-significantly enriched terms, respectively. (**B**) Sankey bubble plot of DEGs enriched in hormone signal transduction pathways (highlighted in red in **A**). Pathway names (such as hormone signaling, cell cycle, sugar metabolism) are labeled in red. In both (**A**,**B**), bubble size corresponds to gene count, while color intensity represents enrichment significance based on −log_10_ (Q value). (**C**) Expression patterns of DEGs associated with auxin biosynthesis and signaling pathways. (**D**) Expression patterns of DEGs involved in cytokinin biosynthesis and signaling pathways. (**E**) Expression profiles of key genes in the strigolactone pathway. In heatmaps, red and blue shades denote up- and down-regulation, respectively. DEGs were filtered with |log_2_ (fold change)| ≥ 2 and Q value ≤ 0.05.

To understand the biological processes underlying transcriptome changes after decapitation, we performed KEGG enrichment analysis on all DEGs identified at different time points (Figure 2A; Table S1B). The DEGs were mainly enriched in pathways related to metabolism and signal transduction. These included metabolic pathways, biosynthesis of secondary metabolites, phenylpropanoid biosynthesis, photosynthesis, sesquiterpenoid and triterpenoid biosynthesis, flavonoid biosynthesis, nitrogen metabolism, and plant hormone signal transduction. Notably, 254 DEGs were enriched in the plant hormone signaling pathway (Figure 2A; Table S1B), suggesting that hormone signaling plays a significant role in the early response to decapitation.

We further conducted GO enrichment analysis of these 254 hormone-related DEGs (Figure 2B). A total of 227 genes were annotated to 100 biological process terms (GO_P), among which 48 were significantly enriched (Table S1C). These GO terms included developmental processes such as floral meristem determinacy, floral organ morphogenesis, stamen development, fruit morphogenesis, seed development, and adaxial–abaxial polarity establishment. Several hormone-related signaling processes were also enriched, including auxin-activated signaling pathway, auxin homeostasis, and responses to cytokinin, gibberellin, and abscisic acid. In addition, terms related to unidirectional cell growth, cell cycle G1/S transition, sucrose response, and light stimulus response were also enriched (Figure 2B; Table S1C). These results indicate that axillary buds activate a multilayered biological response after decapitation, involving complex signal transduction, organogenesis, cell growth, and environmental adaptation. These biological processes are all regulated by hormone signaling pathways.

The results demonstrate that hormone signals, including auxin, cytokinin, and gibberellin, play a central role in dormancy release and bud outgrowth. They function by coordinating with external cues such as sugars and light to regulate the early dynamic development of axillary buds.

### 2.3. Transcriptomic Changes in Hormone Biosynthesis and Signaling Pathways

To clarify the regulatory role of plant hormones in axillary bud development after decapitation, we systematically analyzed hormone-related differentially expressed genes (DEGs). The results showed that key genes involved in both hormone biosynthesis and signaling pathways exhibited significant changes at early time points. As shown in Figure 2C, in the auxin (IAA) pathway, genes encoding core biosynthetic enzymes such as *TAR*, *CYP83B1*, *NIT*, and *YUCCA* were downregulated at 1 h post-decapitation. Genes involved in signal transduction, including *TIR1* (receptor), *ARF* (auxin response factor), and some *SAUR* (small auxin upregulated RNAs), were also downregulated. However, certain members, such as *AUX*/*IAA* (*LOC107774041*) and *SAUR* (*LOC107795057*), were already upregulated at 0.5 h (Table S1D). These data indicate that in the extremely early stage of decapitation, auxin signaling was rapidly reduced. This reduction likely weakens its inhibitory effect on the buds, thereby triggering their release from dormancy. In addition, genes such as *TSB* (tryptophan synthesis), *CYP79B2*, *AUX/IAA*, and *GH3* were upregulated at 6 h and later, suggesting their roles in promoting bud transition to active growth.

In the CK pathway, several biosynthetic enzymes (*IPT*) and degradation enzymes (*CKX*) were upregulated at 1 h post-decapitation (Figure 2D; Table S1D). Simultaneously, key signaling components—including the receptor *CRE1*, phosphotransfer proteins (*AHPs*), and response regulators (*A-ARRs*, *B-ARRs*)—were also induced. Meanwhile, they and CK-activating enzyme *LOG* were also significantly upregulated at 12 h and later. It indicated that CK signaling was activated slightly later than auxin but also contributed to dormancy release and bud development.

Some of the genes involved in the GA-related pathway (such as *CPS*, *KAO*, *GA20ox*) and the negative regulatory factors DELLA, etc., exhibited a response at 1 h after decapitation. Some key genes that promote synthesis and signal transduction were upregulated at 6 h and later (Figure S1A, Table S1D). It is indicated that GA responds to decapitation induction and plays an important role in the continued growth of axillary buds after they break dormancy. Its expression pattern resembles that of CK, indicating a possible synergistic role in bud activation. In contrast, ABA biosynthetic genes *ABA4* and *NCED3* were downregulated at 1 h, while key signaling components such as *PYL* and *PP2C* were upregulated after 6 h (Figure S1B; Table S1D). These results indicate that an early reduction in ABA signaling may help release bud dormancy.

Strigolactone (SL), a newly recognized hormone involved in bud development, also showed early changes. Expression of its biosynthesis genes (*PSY*, *CCD4*, *D27*, *D14*, *MAX2*) and downstream signal integrator *BRC1* was downregulated starting at 1 h. While the expression of suppressors such as *TPRs* was upregulated. Significant changes in all of them were observed at 12 h (Figure 2E; Table S1D). This indicates that SL signaling decreased rapidly and continued to decline, thereby reducing inhibition on axillary buds. Furthermore, the SL-downstream BRC1 network further suppressed ABA-mediated inhibition. For instance, its downstream target *HB40* was significantly down-regulated starting at 6 h. In contrast, the aforementioned *NCED3* exhibited a sustained down-regulation as early as 1 h after decapitation. Together, these results show that multiple hormone pathways were rapidly activated or repressed after decapitation. Auxin responded earliest, and likely released bud inhibition. CK and GA acted synergistically to promote bud activation and growth. Meanwhile, the decline in SL and ABA signals supported the removal of repression. These findings indicate that the transcriptional response of axillary buds to decapitation is governed by a complex hormone interaction network.

### 2.4. Transcriptomic Changes in Nutrient and Light Signaling Pathways

As mentioned previously, the KEGG pathways were enriched in photosynthesis and nitrogen metabolism. To investigate the involvement of nutrient and light signals in dormancy release, we further analyzed the transcriptional expression pattern of DEGs related to these pathways (Figure S1C–F, Table S1E).

In the nitrogen (N) metabolism pathway, plants absorb nitrate (NO_3_^−^) via nitrate transporters (NRT), which is then reduced to nitrite by nitrate reductase (NR), and further incorporated into organic forms via glutamine synthetase (GS) [72,73]. These organic nitrogen compounds are converted into stable asparagine by asparagine synthetase (ASN) [74] and transported to various organs by amino acid permeases (AAP) [75]. Transcriptomic data showed that within 1 h after decapitation, some *GS*, *AAP*, *NR2*, and *ASN1* genes were upregulated. At 12 h, their expression levels increased significantly (Figure S1C; Table S1E). Similarly, key genes involved in phosphorus (P) and potassium (K) signaling pathways were also rapidly induced post-decapitation and showed expression patterns similar to those in the nitrogen pathway (Figure S1D,E; Table S1E). These results suggest that axillary buds received a rapid supply of essential nutrients—particularly N, P, and K—from the main plant body. This nutrient influx provides the material basis for dormancy release and the initiation of growth.

In terms of light signaling, *PHYB* (phytochrome B) was slightly upregulated at 1 h, while the far-red light receptor *FAR1* was slightly downregulated (Figure S1F; Table S1E). It indicated an increase in the perceived red/far-red (R/FR) light ratio. A higher R/FR ratio promotes bud release by alleviating light-mediated suppression, which may further enhance branching.

In summary, during the early response to decapitation, axillary buds not only underwent hormonal reprogramming but also received enhanced nutrient transport and light signal modulation. These changes work together to support the transition from dormancy to active growth.

### 2.5. Prediction and Analysis of Transcription Factors

Transcription factors (TFs) play key roles in organogenesis and cell fate determination in plants. To investigate their roles during axillary bud activation, we analyzed TF-related DEGs after decapitation. A total of 20 transcription factor families were identified with significant differential expression. These included typical developmental regulators such as NAC, MYB, WRKY, KNOX, GATA, and GRAS (Figure S2, Table S1F).

**Figure 3:**
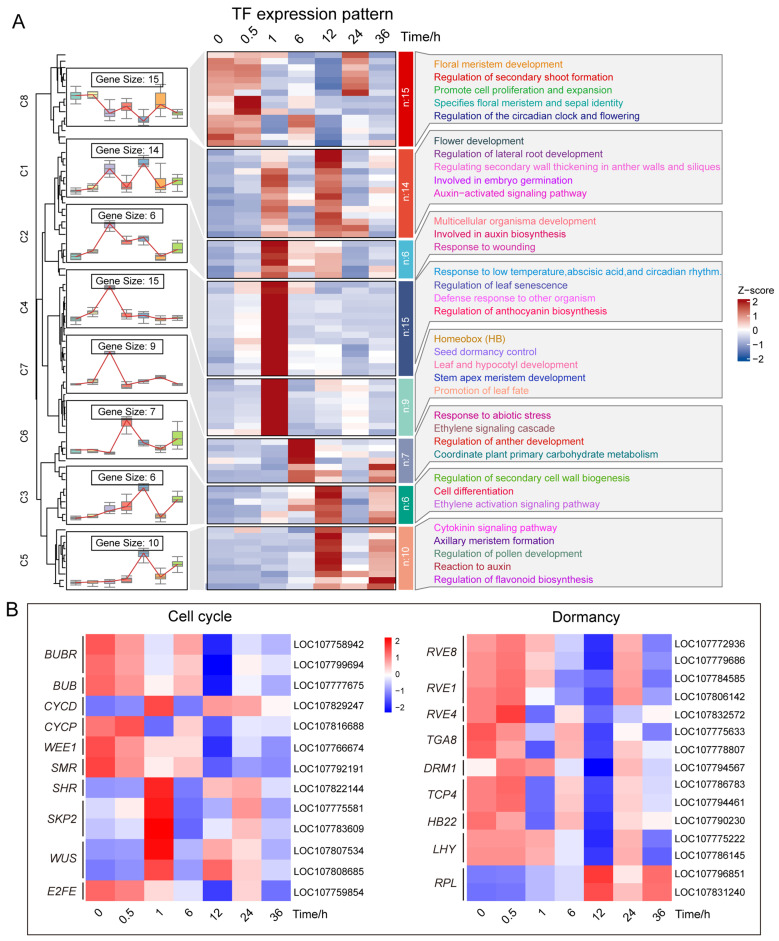
Predictive analysis of development-related regulators among DEGs. (**A**) Expression patterns and enrichment analysis of transcription factors (TFs) after decapitation. From left to right: C, cluster, expression trends of TFs, heatmap and GO/Pathway functional annotations of TFs. (**B**) Expression patterns of DEGs associated with the cell cycle pathway and dormancy-related genes. In heatmaps, red and blue shades denote up- and down-regulation, respectively. DEGs were filtered with |log_2_ (fold change)| ≥ 2 and Q value ≤ 0.05.

Functional annotation revealed that these TFs were mainly associated with developmental processes. Such as embryonic germination, cell differentiation, axillary meristem formation, leaf and hypocotyl development, shoot and floral meristem activity, leaf fate determination, secondary bud regulation, and cell proliferation and expansion (Figure 3A; Table S1F). Some TFs were also linked to hormone signaling pathways, including auxin and cytokinin response. Expression heatmaps showed that many key TFs were significantly upregulated as early as 1 h after decapitation. This indicates that multiple regulatory networks were rapidly activated in axillary buds. The dynamic expression of these TFs reflects the transition from dormancy to differentiation and highlights the broad developmental impact of decapitation on plant systems.

### 2.6. Transcriptomic Changes in Cell Cycle and Dormancy-Related Pathways

Because some of the transcription factors differentially expressed after decapitation were annotated to cell proliferation and differentiation processes. Therefore, we further focused on the expression patterns of DEGs related to cell cycle regulation (Figure 3B; Table S1G). The results showed that, at the very early stage (1 h), many key cell cycle genes exhibited significant changes in expression. Specifically, several negative regulators of the cell cycle, such as *WEE1*, *BUB*/*BUBR*, *CYCP*, *E2FE*, and *SMR*, were downregulated at 1 h after decapitation. In contrast, positive regulators, including *CYCD*, *SHR*, *SKP2*, and the proliferation-related gene *WUS*, were significantly upregulated at the same time point. These transcriptional changes suggest that decapitation rapidly initiated the cell cycle process, priming axillary bud cells for proliferation.

At the same time, we observed clear temporal patterns in the expression of genes related to dormancy signaling (Figure 3B; Table S1G). For instance, the genes encoding transcription factors *TGA8*, *TCP4*, *HB22*, and *RVE4* were significantly down-regulated at 1 and 12 h after decapitation. The circadian rhythm-related genes *RVE8*, *RVE1*, and *LHY* were significantly down-regulated at 6 and 12 h after decapitation. This suggests that decapitation may have disrupted the circadian rhythm of the dormancy maintenance mechanism. Conversely, the growth marker gene *RPL* was upregulated starting at 6 h and peaked at 12 h, suggesting its involvement in the subsequent bud activation process. Notably, *DRM1*, a well-established marker gene for axillary bud dormancy, showed a biphasic expression pattern. It was upregulated at 0.5 h and 1 h, then began to decrease at 6 h and reached its lowest level at 12 h.

These results demonstrate that axillary buds rapidly activate both cell cycle and dormancy-related signaling pathways after decapitation. Such early molecular events suggest that buds accelerate cellular activity and overcome dormancy barriers, laying the groundwork for rapid outgrowth and differentiation.

### 2.7. Enrichment Analysis of DEGs at the Early Response Stage (0.5–1 h)

**Figure 4:**
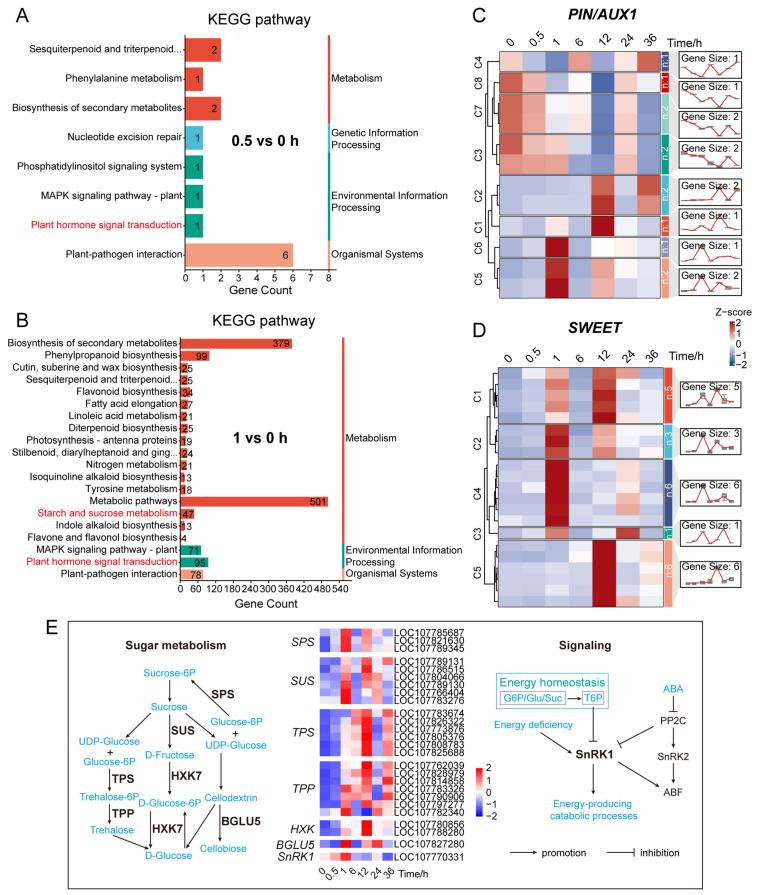
Expression changes of DEGs at the very early stage after decapitation. (**A**,**B**) KEGG pathway enrichment analysis of DEGs at 0.5 h and 1 h after decapitation, respectively. Hormone- and sugar metabolism-related pathways are highlighted in red. (**C**) Expression patterns of auxin transporter genes *PIN/AUX1* across different time points after decapitation, shown as a heatmap and expression trend lines; C, cluster. (**D**) Expression patterns of sugar transporter genes *SWEETs*, presented as a heatmap and expression trend lines; C, cluster. (**E**) Expression profiles of key genes involved in sugar metabolism and related signaling pathways. In all heatmaps, red and blue shades represent up-regulated and down-regulated genes, respectively. DEGs were identified using thresholds of |log_2_ (fold change)| ≥ 2 and Q value ≤ 0.05.

We sought to investigate how differentially expressed genes (DEGs) contribute to the transition of axillary buds from dormancy to growth during the early response to decapitation. Therefore, we performed a systematic comparison and pathway enrichment analysis of DEGs at 0.5 h and 1 h post-decapitation (Figure 4A,B and Figure S3). This analysis aimed to reveal the earliest molecular regulatory networks and provide insight into the mechanism of bud dormancy release.

At 0.5 h after decapitation, DEGs were mainly enriched in pathways related to phenylalanine metabolism, plant hormone signal transduction (particularly auxin response), and secondary metabolite biosynthesis (Figure 4A and Figure S3B; Table S1H). The activation of these pathways suggests that plants rapidly initiated hormone signaling and perception mechanisms at the very beginning of decapitation-induced response (Figure 2C, Figure 4A and Figure S3B), thereby laying the foundation for subsequent developmental transitions.

At 1 h post-decapitation, in addition to continued enrichment in the above signaling pathways, DEGs were significantly enriched in multiple carbohydrate metabolism-related pathways. These included sucrose metabolism, trehalose biosynthesis, glycogen and starch biosynthesis, UDP-glucose metabolism, and cellulose catabolism (Figure 4B and Figure S3A,C; Table S1I). The concentrated activation of these sugar metabolic pathways indicates that axillary buds had quickly entered a phase of energy regulation and material transformation. This provided essential energy and structural resources to support cell division and organ regeneration.

Together, these findings demonstrate that axillary buds undergo a rapid shift from signal perception (via hormone responses) to energy mobilization (via carbohydrate metabolism) within the first hour following decapitation. This transcriptional transition suggests that dormancy release requires not only fast hormonal regulation but also synchronized sugar metabolism, which jointly support the reactivation of bud growth.

### 2.8. Transcriptional Profiling of Sugar Metabolism-Related Genes

Sugars are one of the primary energy sources supporting plant growth and development. Previous studies have shown that sucrose not only functions in metabolic pathways but may also act as a secondary messenger of auxin, mediating apical dominance over axillary buds. In our previous GO enrichment analysis of DEGs and KEGG pathway enrichment analysis of DEGs at early stage of decapitation, several genes were enriched in the biological process “response to sucrose” (Figure 2B) and the “starch and sucrose metabolism” pathway (Figure 4B; Table S1I). It suggested that sugar metabolism may play a key role in the release of bud dormancy.

To explore this further, we analyzed the transcriptional expression patterns of key genes involved in sugar metabolism (Figure 4E; Table S1J). The results showed that genes encoding sucrose phosphate synthase (*SPS*), sucrose synthase (*SUS*), β-glucosidase (*BGLU5*), and trehalose-6-phosphate phosphatase (*TPP*) were significantly upregulated at 1 h after decapitation. In contrast, genes encoding trehalose-6-phosphate synthase (*TPS*) and hexokinase (*HXK*) showed significant upregulation at 12 h. Sucrose non-fermenting-related protein kinase 1 (*SnRK1*), as the “energy sensor” and “energy regulation center” of plant cells [76,77,78,79,80], was significantly upregulated one hour after decapitation. These results indicate that sugar metabolism was initiated very early after decapitation and was further enhanced at later stages. This early activation provided sufficient energy to support the transition of axillary buds from a quiescent to a growth-active state.

To further uncover the coordination between sugar and hormone signaling, we analyzed the expression patterns of auxin and sugar transporter-related genes (Figure 4C,D; Table S1K). Several *PIN* genes (Figure 4C cluster4), which encode polar auxin transporters, were downregulated at 0.5 h and significantly downregulated at 1 h, while others showed upregulation at 1 h or 12 h. Meanwhile, sugar transporter genes from the SWEET family were significantly upregulated at both 1 h and 12 h. Together with the enrichment of sugar metabolic pathways at 1 h, these results suggest that auxin homeostasis in axillary buds was disrupted first, followed by rapid sugar accumulation and active metabolism, which jointly promoted dormancy release.

Overall, decapitation rapidly induced the activation of sugar biosynthesis and metabolism pathways, accompanied by increased expression of sugar transporters and changes in auxin transport dynamics. These results suggest a dynamic shift in the balance between sugar and auxin signaling. We propose that bud dormancy release may depend on local thresholds of sugar and auxin concentrations and the strength of their signaling. This mechanism likely represents a key regulatory node in axillary bud activation and branch initiation.

### 2.9. Transcriptomic Dynamics and qRT-PCR Validation of DEGs Involved in Axillary Meristem Initiation

**Figure 5:**
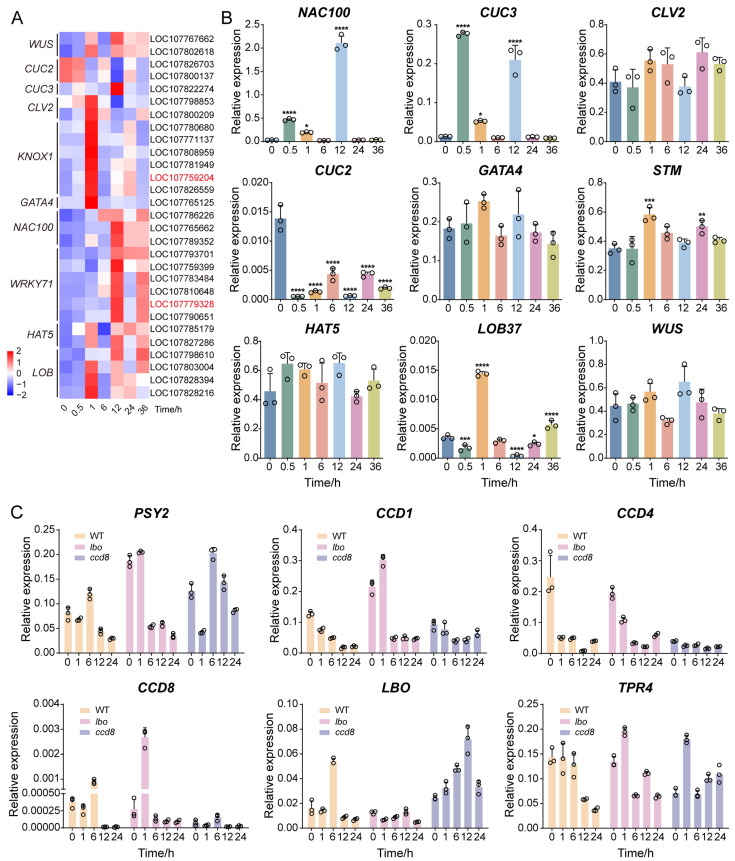
Expression patterns of axillary bud development-related DEGs and qRT-PCR validation. (**A**) Transcriptomic expression profiles of DEGs associated with axillary bud initiation and development. Red and blue shades represent up-regulated and down-regulated genes, respectively. Red labels indicate the candidate knockout genes (*LCO107759204* and *LOC107779328*). (**B**) qRT-PCR results of nine selected candidate genes. (**C**) qRT-PCR analysis of key strigolactone pathway genes in wild-type (WT) and mutant plants (*lbo*, *ccd8*) at different time points (0, 1, 6, 12, 24 h) after decapitation at the eight-leaf stage. Error bars indicate standard error (*n* = 3). Asterisks denote statistically significant differences (* *p* < 0.05, with significant correlation; ** *p* < 0.01, *** *p* < 0.001 and **** *p* < 0.0001, with extremely significant correlation; One-way ANOVA followed by Dunnett’s test was applied.). The circles in the bar plots represent each data point. For A, DEGs were identified using thresholds of |log_2_ (fold change)| ≥ 2 and Q value ≤ 0.05.

TF prediction analysis revealed that DEGs were significantly enriched in axillary meristem pathways. Therefore, we analyzed the expression of key genes to determine how decapitation affects axillary bud initiation (Figure 5A; Table S1L).

The results showed that several essential genes—including *WUS*, *CLV2* and *KNOX1* (maintaining the homeostasis of the shoot apical meristem), *LOB* and *CUC3* (lateral organ boundary development)—were upregulated as early as 1 h after decapitation. Meanwhile, *CUC2* was down-regulated (Figure 5A). These findings suggest that decapitation rapidly activated genes associated with axillary meristem initiation. These genes are known to interact with hormone signaling pathways to form a complex regulatory network that controls the initiation and development of axillary meristems. Combined with the observed changes in hormone signaling and transcription factor expression, we infer that decapitation disrupts the “Hormone–TF” regulatory network that maintains bud dormancy and triggers the initiation of axillary bud development.

To validate the transcriptomic data, we selected 9 representative genes for qRT-PCR analysis. These included seven genes related to axillary meristem initiation (*NAC100*, *CUC3*, *CLV2*, *CUC2*, *STM*, *LOB37*, *WUS*), and two transcription factors (*GATA4*, *HAT5*) (Figure 5B). The results showed significant upregulation or downregulation of these genes at various time points after decapitation. Most genes exhibited expression changes as early as 0.5 h or 1 h, and their expression trends were consistent with the RNA-seq results.

These data confirm that decapitation rapidly activates the expression of key regulators involved in axillary bud initiation. For further experimental validation, we selected *LCO107759204* (*STM*) from the KNOX1 family and *LOC107779328* (*EXB1*) from the WRKY family as knockout candidates.

### 2.10. Transcriptomic Analysis and Mutant Validation of SL Pathway Genes Involved in Axillary Bud Development

To further validate the role of strigolactone (SL) signaling pathway during the development of axillary bud induced by decapitation, we selected six SL-related genes (*PSY2*, *CCD1*, *CCD4*, *CCD8*, *LBO* and *TPR4*) for functional validation using available mutant lines. Specifically, *ccd8* and *lbo* mutants were subjected to decapitation at the 8-leaf stage, and axillary bud development was observed (Figure S4). The results showed that visible morphological changes in axillary buds occurred within 24 h after decapitation in both mutants and wild-type (WT) plants. However, the growth rate of buds in the mutants was significantly faster than that in WT. This suggests that the SL signaling pathway plays an inhibitory role in bud growth. And that reduced or absent SL signaling in the mutants relieves this inhibition, thereby accelerating bud development.

To explore the timing of this response, we further examined the expression patterns of the selected SL pathway genes at five time points (0, 1, 6, 12, and 24 h) following decapitation in both WT and mutant plants (Figure 5C). In WT plants, most genes showed significant expression changes as early as 1 h after decapitation. Similar trends were observed in the mutants. However, due to the functional loss of *ccd8* and *lbo*, expression patterns of some SL-related genes were altered.

These results are highly consistent with our transcriptome data and confirm that SL pathway genes respond to external stimuli at a very early stage (1 h post-decapitation). This supports the conclusion that the SL pathway is involved in dormancy release and developmental activation of axillary buds. In summary, our findings demonstrate that decapitation rapidly activates hormone regulatory pathways related to axillary bud development. In particular, the inhibitory role and temporal specificity of the SL pathway in dormancy release are key regulatory factors for reinitiating bud growth.

### 2.11. Differentially Expressed Gene NtSTM Regulates Axillary Bud Growth and Influences Multi-Organ Development in Tobacco

**Figure 6:**
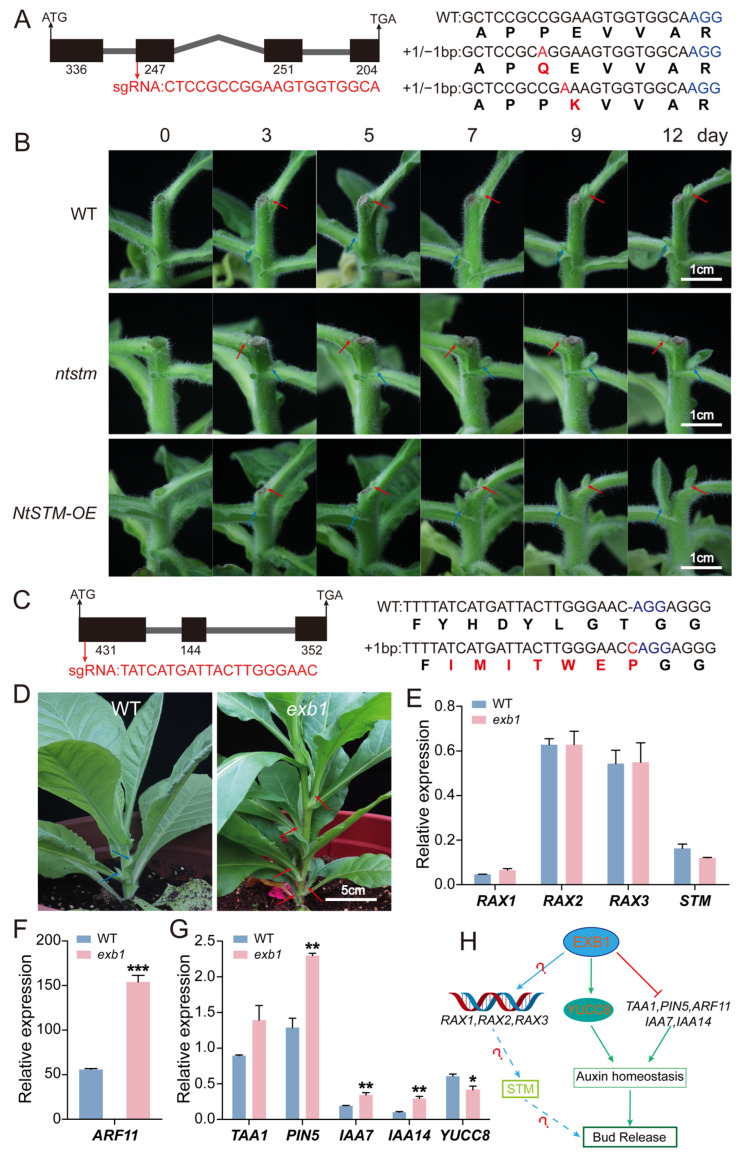
Functional validation of DEGs *NtSTM* and *NtEXB1*. (**A**) Gene structure of *NtSTM*, with target sites highlighted in red and base-editing mutations in mutant alleles indicated (red letters). The bold letters represent the amino acids corresponding to the bases. (**B**) Phenotypes of control (WT), mutant (*ntstm*), and overexpression (*NtSTM-OE*) transgenic tobacco lines at the seven-leaf stage following decapitation. Images were taken at 0, 3, 5, 7, and 9 days after treatment. Red and blue arrows indicate axillary buds at the first and second leaf positions, respectively. Scale bar, 1 cm. (**C**) Gene structure of *NtEXB1*, with target sites (red) and base-editing mutations (red letters) shown. The bold letters represent the amino acids corresponding to the bases. (**D**) Phenotype of the *ntexb1* mutant. Blue and red arrows mark axillary buds in WT and *ntexb1* mutants, respectively. Scale bar, 5 cm. (**E**–**G**) Transcript levels of putative *EXB1* downstream genes in the *ntexb1* mutant. (**H**) Proposed model illustrating the role of *NtEXB1* in regulating branching development. Error bars represent standard error (n = 3). Asterisks indicate statistically significant differences (* *p* < 0.05, with significant correlation; ** *p* < 0.01 and *** *p* < 0.001, with extremely significant correlation; Student’s *t*-test).

Transcriptome analysis revealed that several members of the KNOX1 transcription factor family were rapidly activated at the early stage following decapitation. To further investigate their functions, we selected an axillary bud development marker gene *SHOOT MERISTEMLESS* (*STM*, *LOC107759204*), that was rapidly upregulated at the early stage of decapitation with high expression in the stem, for functional studies (Figure 5A, Figure 6A,B and Figure S5). The full-length CDS of *NtSTM* (1038 bp) was cloned, comprising four exons and encoding a protein with the conserved KNOX1 and KNOX2 domains typical of the KNOX family (Figure 6A and Figure S6A). Tissue expression profiling confirmed that *NtSTM* was highly expressed in stems, consistent with transcriptomic results (Figures S5 and S6B).

We used CRISPR/Cas9 technology to target the second exon of *NtSTM* and obtained heterozygous mutants carrying a single base substitution. These mutants showed no significant differences in overall plant morphology compared to wild-type (WT) plants (Figure 6A and Figure S6C). In parallel, we generated *NtSTM* overexpression lines driven by the 35S promoter. Compared with WT, overexpression plants exhibited distinct morphological abnormalities in leaves, including severe curling and wrinkling (Figure S6D,F).

To explore the regulatory role of *NtSTM* in axillary bud development, we performed decapitation on WT, *NtSTM* mutants, and overexpression plants at the seven-leaf stage. And the development of axillary buds was subsequently monitored at 0, 3, 5, 7, 9, and 12 days post-decapitation (Figure 6B). The results showed that by day 3, visible changes appeared in the first-node axillary buds across all genotypes. In WT, only the first-node buds continued to grow, while second-node buds remained suppressed. However, in both *NtSTM* mutants and overexpression lines, second-node buds grew faster than first-node buds starting from day 5. Notably, in the mutants, growth of the first-node buds was significantly inhibited. By day 12, axillary buds in overexpression lines showed faster growth at the same nodes compared to both WT and mutants, whereas mutants exhibited delayed development at the first node. These findings suggest that *NtSTM* not only affects bud growth but also plays a differential regulatory role across nodal positions.

In addition, *NtSTM* affected the development of floral organs and chloroplast structure. Compared with WT, *NtSTM* overexpression plants displayed altered floral morphology, including thinner corollas, larger petal indentation angles, more rounded petal tips, smaller flower size, and reduced plant height (Figure S6E,F). Microscopic observation revealed that chloroplasts in overexpression leaves were structurally disorganized, with abnormal stacking of grana thylakoids and disrupted grana morphology (Figure S6G). These structural defects may lead to reduced photosynthetic efficiency, thereby affecting overall plant growth.

In conclusion, *NtSTM* plays a key role in regulating axillary bud development. Moreover, it contributes—either directly or indirectly—to the formation of floral organs and the structural integrity of chloroplasts, thus influencing the morphogenesis of multiple plant organs.

### 2.12. Differentially Expressed Gene NtEXB1 Regulates Axillary Bud Development by Modulating Auxin Homeostasis

Previous studies have shown that EXB1, a member of the WRKY transcription factor family in Arabidopsis thaliana, acts upstream of *STM* and plays a key regulatory role in axillary meristem initiation. To explore its potential function in tobacco, we identified a differentially expressed gene, *NtEXB1* (*LOC107779328*), from our transcriptome dataset. This gene showed low expression in dormant axillary buds but was significantly upregulated at 12 h after decapitation (Figure S5 and Figure 5A). These results suggest that *NtEXB1* may respond to decapitation and be involved in the regulation of axillary bud development in tobacco. Based on the Arabidopsis database and NCBI sequence information, we cloned the full-length CDS of *NtEXB1* (927 bp), which consists of three exons (Figure 6C). Conservation analysis of the functional domain and amino acid sequence alignment confirmed that NtEXB1 contains a typical WRKY domain and shares high similarity with AtEXB1 (Figure S7), suggesting that they may have similar functions.

We employed the CRISPR/Cas9 system to target the first exon of *NtEXB1* for knockout. This generated an *ntexb1* mutant carrying a frameshift mutation caused by a single-base insertion (Figure 6C,D). Phenotypic observation during the rosette stage revealed that *ntexb1* mutants developed five visible axillary buds per plant, each longer than 3 cm, with three buds exceeding 5 cm. This phenotype indicates that apical dominance was released and these buds had the potential to develop into branches. In contrast, axillary buds in wild-type plants were generally shorter than 2 cm and remained suppressed by apical dominance.

To further investigate the regulatory pathway of *NtEXB1*, we examined the expression levels of *RAX* genes, *STM*, and auxin-related genes in the mutant (Figure 6E–G). The expression of *RAX1*, *RAX2*, *RAX3*, and *STM* remained unchanged compared with the wild type. However, several auxin-related genes—including the biosynthesis gene *TAA1*, transporter *PIN5*, and signaling components *IAA7*, *IAA14*, and *ARF11*—were significantly upregulated, while *YUC8* was downregulated. These results indicate that auxin biosynthesis and signaling were strongly perturbed in the ntexb1 mutant. In contrast, the downstream RAXs–STM regulatory module for bud initiation remained unaffected.

Collectively, these data suggest that *NtEXB1* indirectly influences axillary bud development by modulating auxin homeostasis, rather than acting directly through the RAXs–STM pathway (Figure 6H). The transcriptome data showing its significant upregulation at 12 h after decapitation further support the involvement of *NtEXB1* in regulating bud outgrowth after dormancy release. This also implies that the release of dormancy likely occurs before 12 h post-decapitation.

## 3. Discussion

Decapitation promotes the release of axillary bud dormancy and their transition to branching [13]. This study demonstrates that the release of dormancy in tobacco axillary buds induced by decapitation is initiated at an extremely early stage—within 0.5 to 1 h post-treatment. Consistent with the findings of Weifeng Wang [70] and Lin Wang [69] et al., we observed macroscopic enlargement of axillary buds by 24 h post-decapitation. Concurrently, the expression levels of key genes in hormone and sugar signaling pathways likewise exhibited significant changes. In the study by Lin Wang et al., the IAA content decreased at 12 h after decapitation but increased by 24 h, whereas the CK content progressively increased. Concurrently, the levels of both GA and ABA exhibited a continuous decrease [69]. Similarly, our study revealed that during the late phase (12–24 h) after decapitation, the expression of key IAA biosynthetic genes was down-regulated at 12 h but up-regulated at 24 h, while that of key CK biosynthetic genes was consistently up-regulated. Meanwhile, key ABA biosynthetic genes were continuously down-regulated, whereas key GA biosynthetic genes showed decreased expression at 24 h. We also observed that the expression patterns of key genes across several hormone signaling pathways mirrored those found by Lin Wang. Furthermore, in this study, the bud dormancy marker gene *DRM1* exhibited a sustained down-regulation at 6 and 12 h after decapitation. This expression pattern is consistent with the reported decline of *DRM1* in Arabidopsis between 6 and 12 h post-decapitation [81], and also aligns with its protein expression dynamics in pea [82], where levels are high at 1–3 h but drop rapidly after 6 h. In contrast, our work identified significant alterations in the expression of numerous genes linked to hormone, sugar, cell cycle, and dormancy pathways within the first 0.5–1 h. This rapid response parallels the observations of Da Cao et al., wherein a decline in IAA and a rise in CK content were detected by 3 h after decapitation [58]. Furthermore, our data support the earlier observation by Franziska Fichtner et al. of a rise in trehalose 6-phosphate concentration at 2 h following shoot apex removal [83]. The mutual confirmation between our discoveries and these known findings serves to validate the accuracy of our experimental data and conclusions. Consequently, our findings lead us to conclude that the axillary bud fate transition from dormancy to growth is not a slow, cumulative process, but rather a rapid and decisive event. Therefore, prior studies focusing on responses several hours or days after decapitation [71,84] may have primarily captured post-dormancy developmental progression, rather than the initiation phase. Our early-stage transcriptomic analysis fills this critical knowledge gap.

Our findings further reveal that disruption of auxin homeostasis and enhancement of sugar signaling serve as key triggers for dormancy release. This conclusion differs from the prevailing models that attribute the initial trigger to sugar signaling [48] or to cytokinin-sugar synergy [58]. Our study reveals that genes involved in auxin signaling began to respond as early as 0.5 h, with significant downregulation observed at 1 h. While sugar transport and metabolism genes were significantly upregulated at 1 h. This supports the idea that auxin and sugar are core initiators of dormancy release. Given that auxin is a known suppressor of axillary bud growth [17,85], while sugars are essential energy sources for bud outgrowth [48], we propose a two-step “signal–energy” model. Auxin signaling responds to decapitation by rapidly adjusting synthesis, transport, and homeostasis, leading to a transient decrease in local auxin levels and release of inhibitory signals. This is followed by sugar accumulation and metabolic activation to fuel the re-initiation of growth. However, this conclusion currently rests solely on transcriptomic data. Future validation through integrated proteomic and metabolomic analyses, along with expanded sample sets and more rigorous experimental controls, is required. Nevertheless, this “signal–energy” model provides a new perspective for understanding dormancy release and suggests future directions using metabolomics and fluorescent hormone sensors to capture dynamic changes in real time.

Our transcriptomic data also revealed a rapid and comprehensive reprogramming of the regulatory network underlying bud development after decapitation. Multiple signaling pathways—including IAA, CK, SL, ABA, and sugars—exhibited significant transcriptional changes at the early stage. These alterations align with their established functions: IAA suppresses bud growth [19], SL and ABA reinforce dormancy [86,87], while CK, GA, and sugars promote branching [37,40,88]. Moreover, key transcriptional regulators of axillary meristem initiation, such as *CUC* [89], *RAX* [90], *LOB* [91], *KNOX* [92], *CLV* [93], and *WUS* [94], were also activated within 1 h, suggesting their involvement in the dormancy release program. These findings indicate that dormancy release is not an isolated event, but rather a result of system-wide regulatory imbalance and re-establishment. This rapid network reconfiguration of developmental control may allow the plant to quickly redirect growth following the loss of apical dominance.

Phenotypic observations of *NtSTM* mutants and overexpression lines further support its role in regulating axillary bud initiation. Although *NtSTM* was not completely knocked out, changes in its expression levels significantly affected the development of first-node buds and overall plant morphology (e.g., dwarfing, leaf curling, altered floral traits). These findings indicate that *NtSTM* not only maintains axillary bud [95] but also broadly regulates plant morphology, possibly through regulation of cell differentiation and hormone responses. Functional analysis of *NtEXB1* revealed that its expression was significantly upregulated at 12 h post-decapitation. The *ntexb1* mutant exhibited multiple, elongated axillary buds with enhanced branching potential, indicating that *NtEXB1* regulates bud growth after dormancy release. Unlike in Arabidopsis, where EXB1 activates *RAX* genes to initiate bud formation [96], *RAX* expression in the *ntexb1* mutant remained unchanged. Instead, significant changes were observed in auxin biosynthesis, transport, and signaling genes. These results suggest that *NtEXB1* regulates bud development indirectly by modulating auxin homeostasis. This highlights both the conserved and species-specific aspects of regulatory mechanisms and underscores the central role of WRKY transcription factors in the axillary bud regulatory network.

**Figure 7:**
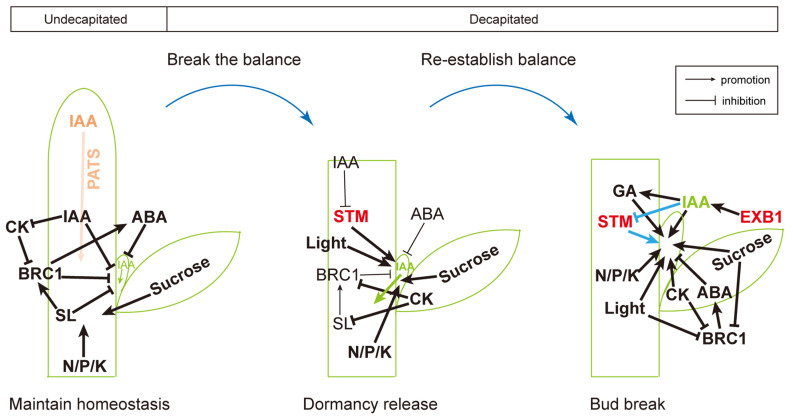
A proposed regulatory network model for decapitation-induced axillary bud transition from dormancy to release and subsequent branch development in tobacco. Schematic representation of stem architecture (green): shoot apex, stem, leaf axil, and axillary bud. States from left to right: undecapitated, bud release after decapitation, and bud outgrowth post-decapitation. The blue curved arrow indicates the transition following shoot apex removal. Orange text and arrow denote IAA synthesized in the shoot apex and its basipetal transport via the polar auxin transport stream (PATS). Green text and arrow represent IAA produced in the axillary bud and its export into the stem. Black labels within the stem indicate dormancy-associated regulators. Target genes validated in this study are highlighted in red (*STM*, *EXB1*). Blue arrows and lines depict proposed novel regulatory relationships and homeostatic adjustments between IAA, *STM*, and bud development. Line thickness corresponds to the relative strength of the interaction or magnitude of transport.

Based on transcriptomic and genetic findings, we propose a multi-signal integrated model for axillary bud development (Figure 7). After decapitation, the auxin (IAA) supply from the stem apex declines, and the IAA signal in the axillary bud drops temporarily, while SL and ABA signaling are weakened. Concurrently, CK pathways are activated, and sugar levels rise to provide energy. The expression of the key branching inhibitor *BRC1* is downregulated, further lifting repression on buds. As light, nutrient, and hormonal signals are integrated, the bud transitions from dormancy to growth and progressively establishes a new regulatory program for branch development. The dynamic network reprogramming delineates the sequential events of dormancy release and activation, and provides a theoretical basis and potential molecular targets for improving crop branching traits.

## 4. Materials and Methods

### 4.1. Plant Materials

Tobacco cultivar *Nicotiana tabacum* cv. Honghuadajinyuan at the early flowering stage was used in this study. Plants with similar growth status were selected for decapitation. Axillary buds were collected at 0 (control), 0.5, 1, 6, 12, 24, and 36 h post-decapitation. In addition, various tissues, including axillary buds, roots, stems, leaves, stamens, and pistils, were collected at 0 h as control samples. Each sample pool consisted of 3 plants, with three independent biological replicates, yielding a total of 36 samples (see Supplementary Table S1A for details). All samples were immediately frozen in liquid nitrogen and stored at –80 °C for subsequent RNA extraction and library preparation. Tissues including roots, stems, leaves, axillary buds, flowers, stamens, pistils, sepals, petals, anthers, filaments, ovaries, styles, and stigmas were also collected at the full-bloom stage for tissue-specific expression analysis.

Decapitation procedure: At the 12-leaf seedling stage, the top three young leaves were removed. At the early flowering stage, flower buds and inflorescence leaves were removed. Axillary bud tissues from the first and second leaf nodes (numbered from the top downward) were collected.

Wild-type Honghuadajinyuan, *ntccd8*, and *ntlbo* mutant seeds were preserved in our lab. The parental line for the *ntccd8* and *ntlbo* mutants is *Nicotiana tabacum* cv. Honghuadajinyuan. These mutants were generated by CRISPR/Cas9-mediated editing, exhibiting a high-branching phenotype and reduced strigolactone accumulation. Seed germination and seedling growth were conducted in an artificial climate chamber under the following conditions: 26 °C, 75% humidity, 22,000 lux light intensity, 16 h light/8 h dark cycle. Plants at the rosette, early flowering, and full-bloom stages were grown outdoors at latitudes ranging from 29°39′02″ N to 30°03′02″ N with an average temperature of 23 °C.

### 4.2. RNA Extraction

Tissue samples were ground to a fine powder in pre-chilled mortars with liquid nitrogen. Approximately 30 mg of tissue powder was added per 1 mL of lysis buffer containing 2% CTAB (Bio Basic, Markham, ON, Canada) and 2% β-mercaptoethanol in a 2.0 mL tube. The mixture was incubated at 65 °C with shaking (400–1400 rpm) for 15 min and cooled to room temperature. Following centrifugation at 12,000× *g* for 5 min at 4 °C, the supernatant was transferred to a new 2.0 mL tube. Subsequently, chloroform/isoamyl alcohol (24:1, *v*/*v*) was added (200 μL per mL of lysis buffer), followed by vigorous vortexing. The sample was centrifuged at 12,000× *g* for 10 min at 4 °C. The aqueous phase was transferred to a fresh 2.0 mL tube, mixed with an equal volume of phenol/chloroform/isoamyl alcohol (25:24:1, *v*/*v*), and centrifuged again under the same conditions (12,000× *g*, 10 min, 4 °C). This step was repeated using an equal volume of chloroform/isoamyl alcohol (24:1, *v*/*v*). The final aqueous supernatant was carefully transferred to a new 1.5 mL tube, avoiding any interphase material. RNA was precipitated using 2/3 volume of isopropanol and incubated at –20 °C for at least 2 h. The RNA pellet was collected by centrifugation at 12,000× *g* for 20 min at 4 °C, washed with 1 mL of 75% ethanol, and centrifuged at 17,500× *g* for 3 min at 4 °C. After removing the supernatant, the pellet was air-dried (3–5 min) and finally dissolved in 20–200 µL of DEPC-treated or RNase-free water. The quantity and integrity of the total RNA were assessed using an Agilent 2100 Bioanalyzer (Agilent, Santa Clara, CA, USA).

### 4.3. Library Construction and RNA Sequencing

Library preparation is performed using the Optimal Dual-mode mRNA Library Prep Kit (BGI-Shenzhen, China).

(1) Sample QC was performed based on sample quality and experimental requirements. (2) mRNA was enriched using oligo (dT) magnetic beads. (3) Fragmentation of mRNA was carried out with the fragmentation buffer. (4) First- and second-strand cDNA synthesis was conducted. (5) cDNA end repair, A-tailing, and adaptor ligation were performed. (6) PCR amplification of cDNA libraries was performed. (7) Final library quality was verified before sequencing. (8) Libraries were circularized, and linear DNA was digested. (9) Sequencing was performed on the DNBSEQ platform using cPAS technology.

RNA sequencing was carried out by BGI Genomics Co., Ltd. (Shenzhen, China). Sequencing was performed on an MGI DNBSEQ-T7 (BGI-Shenzhen, China) platform with SE50 read length.

### 4.4. RNA-Seq Data Analysis

(1) Data filtering. The sequencing data were filtered with SOAPnuke [97] by removing reads containing sequencing adapters, the reads whose low-quality base ratio (base quality less than or equal to 15) is more than 20% and whose unknown base (‘N’ base) ratio is more than 5%. Afterwards, clean reads were obtained and stored in FASTQ format. (2) Structure variation detection. The clean reads were mapped to the tobacco reference genome (GCF_000715135.1_Ntab-TN90) using HISAT2 [98]. After that, Ericscript (v0.5.5) [99] and rMATS (V4.1.2) [100] were used to detect fusion genes and differential splicing genes (DSGs), respectively. (3) RNA identification. Bowtie2 [101] was applied to align the clean reads to the gene set, in which known and novel, coding and noncoding transcripts were included. (4) Gene quantification differential expression analysis. Gene expression level was calculated by RSEM (v1.3.1) [102]. Essentially, differential expression analysis was performed using the DESeq2 (v1.34.0) [103] with Q value ≤ 0.05 (or FDR ≤ 0.001). DEGs were identified based on |log2FC| ≥ 2 and Q value ≤ 0.05. (5) Gene annotation. To make insights into the phenotype changes, GO (http://www.geneontology.org/, accessed on 22 October 2025) and KEGG (https://www.kegg.jp/, accessed on 22 October 2025) enrichment analysis of annotated differentially expressed gene was performed by Phyper based on Hypergeometric test. The significant levels of terms and pathways were corrected by Q value with a rigorous threshold (Q value ≤ 0.05) (https://bioconductor.org/packages/release/bioc/html/qvalue.html, accessed on 22 October 2025). (6) Data analysis and visualization. The subsequent analysis and data mining were performed on the Dr. Tom Multi-omics Data mining system (https://biosys.bgi.com, accessed on 24 October 2025). Heatmap was plotted by https://www.bioinformatics.com.cn (last accessed on 10 December 2024), an online platform for data analysis and visualization. This analysis was visualized using the R package ClusterGVis_0.1.4 (https://github.com/junjunlab/ClusterGVis, accessed on 24 October 2025).

### 4.5. cDNA Synthesis and qRT-PCR Validation

To verify the RNA-seq results, qRT-PCR was performed to confirm the expression of selected DEGs. The primers were designed based on sequences obtained from the NCBI database (https://www.ncbi.nlm.nih.gov/, accessed on 22 December 2024). Total RNA from full-bloom stage tissues was extracted using TRIzol^®^ reagent (Invitrogen, Cat. #15596-026, Carlsbad, CA, USA), following the manufacturer’s instructions. Reverse transcription was carried out using M-MLV reverse transcriptase (Promega, Cat. #M1701, Madison, WI, USA). QRT-PCR was performed using NovoStart^®^ SYBR qPCR SuperMix Plus (Novoprotein, Cat. #E096, Suzhou, China). Reactions were prepared according to the manufacturer’s instructions and run on a qTOWER3 Real-Time PCR System (Analytik Jena AG, Jena, Germany). The thermal cycling protocol was as follows: 95 °C for 30 s; 40 cycles of 95 °C for 5 s and 60 °C for 30 s; followed by a melt curve stage: 95 °C for 15 s, 60 °C for 30 s, and a continuous ramp to 95 °C with a 15-sec measurement per step.

Relative gene expression was calculated using the 2^−ΔCT^ method [104]. *Elongation factor 1-alpha* (*EF1α*) was used as the reference gene. Primer information is listed in Table S1M. Each experiment included three biological and three technical replicates.

### 4.6. Gene Cloning, Vector Construction, and Tobacco Transformation

The CDS sequences of *NtSTM* (*LOC107759204*) and *NtEXB1* (*LOC107779328*) were amplified using primers designed based on sequences obtained by aligning our transcriptome data with the NCBI database (see Supplementary Table S1M). Total RNA was extracted from stems and leaves of Honghuadajinyuan, and cDNA synthesis followed the procedures described in Section 4.4. Full-length *NtSTM* and *NtEXB1* CDS were amplified by PCR and cloned into the pCXSN vector under control of the *CaMV-35S* promoter. PCR was performed using GoTaq^®^ Green Master Mix (Promega, Cat. #M7122, Madison, WI, USA) according to the manufacturer’s instructions. Positive PCR products were subsequently cloned into the pEASY-T5 Zero Cloning Vector (TransGene, Cat. #CT501-01, Beijing, China) for sequence verification. For gene knockout, sgRNA sequences targeting the first exon of *NtEXB1* and the second exon of *NtSTM* were designed and inserted into the pORE-Cas9 vector preserved in our lab [105]. Overexpression and knockout constructs were introduced into Agrobacterium tumefaciens strain LBA4404 (WEIDI, Cat. #AC1030, Shanghai, China), and leaf disk transformation was used for tobacco transformation and tissue culture screening [106,107].

### 4.7. Generation of Transgenic Lines

Stable transgenic tobacco lines identified through tissue culture screening were sequenced to confirm the presence of *NtSTM*- and *NtEXB1*-targeting sgRNAs. Positive knockout lines were propagated to the T1 generation. Genomic DNA was extracted from leaf tissue of T1 regenerants using the *EasyPure*^®^ Plant Genomic DNA Kit (TransGene, Cat. #EE112-01, Beijing, China) according to the manufacturer’s protocol. Gene-specific primers (Table S1M) were designed based on the *NtSTM* and *NtEXB1* genomic sequences to amplify regions encompassing the target sgRNA sites (see Section 4.6). The PCR products were subjected to Sanger sequencing, and the resulting sequences were aligned with the wild-type reference to identify mutant lines and characterize the editing profiles. For *NtSTM* overexpression lines, RNA was extracted (See Section 4.4), and qRT-PCR was used to measure gene expression (See Section 4.5). Lines with significantly upregulated *NtSTM* expression were selected for downstream analyses.

### 4.8. Gene Structure and Domain Analysis

The SMART database (http://smart.embl-heidelberg.de/, accessed on 26 October 2025) was used to predict protein domains of NtSTM, NtEXB1, and AtEXB (*AT1G29860*). Amino acid sequence alignment was performed using DANMAN V6.0.3.99 software (Lynnon Biosoft, San Ramon, CA, USA). Gene structure was analyzed using GSDS 2.0 (https://gsds.gao-lab.org/, accessed on 26 October 2025). The PrD core domain was analyzed using PLAAC (http://plaac.wi.mit.edu/, *Lcore* = 60, a = 1, accessed on 26 October 2025).

### 4.9. Transmission Electron Microscopy (TEM)

(1) Fixation: Fresh samples were fixed in 3% glutaraldehyde (Sinopharm Chemical Reagent, Cat. #20230828, Shanghai, China), followed by secondary fixation in 1% osmium tetroxide (EMCN, Cat. #GP18456, Beijing, China). (2) Dehydration: Samples were gradually dehydrated using acetone (XILONG SCIENTIFIC, Cat. #13100201, Shantou, China) in the following series: 30%, 50%, 70%, 80%, 90%, 95%, and 100%, with three exchanges in 100% acetone. (3) Infiltration: Samples were infiltrated with mixtures of dehydrant and Epon-812 (EMCN, Cat. #GS02659, Beijing, China) embedding resin at ratios of 3:1, 1:1, and 1:3. (4) Embedding: Samples were embedded in Epon-812 resin. (5) Sectioning: Ultrathin sections (60–90 nm) were prepared using an ultramicrotome (LEICA, UC7rt, Wetzlar, Germany) and mounted onto copper grids. (6) Staining and imaging: Sections were stained with uranyl acetate (EMCN, Cat. #GS02624, Beijing, China) (10–15 min) and lead citrate (EMCN, Cat. #GA10701-1, Beijing, China) (1–2 min), followed by observation under a transmission electron microscope. Images were acquired using a transmission electron microscope (JEOL, JEM-1400FLASH, Tokyo, Japan).

### 4.10. Statistical Analysis

All statistical analyses were performed using GraphPad Prism10.1.2 (GraphPad Software, CA, USA). Two-tailed Student’s *t*-tests were used for pairwise comparisons. Data are presented as mean ± standard deviation (SD). Significance was defined as *p* < 0.05 (*), *p* < 0.01 (**), *p* < 0.001 (***) and *p* < 0.0001 (****). For multiple group comparisons, one-way ANOVA followed by Dunnett’s test was applied.

## 5. Conclusions

In summary, this study reveals that decapitation-induced axillary bud dormancy release in tobacco occurs at an extremely early stage—within 0.5 to 1 h post-treatment. Auxin, as the primary triggering signal, and sugars, as key energy suppliers, were significantly enriched during this early stage. Along with cytokinin, strigolactone, and abscisic acid, these signals collectively participate in the regulation of dormancy release. Functional gene validation, combined with transcriptomic data, indicates that all kinds of regulatory factors—including plant hormones, sugars, environmental cues (nutrients and light), and transcription factors—jointly govern the dynamic transition from dormancy to bud outgrowth. These factors constitute an interconnected regulatory network in which changes in one element influence others, establishing a new dynamic balance. Given the complexity of axillary bud development and the polyploid nature of the tobacco genome, studying individual factors in isolation may be insufficient to fully understand their role or address practical agricultural needs. A systems-level approach that integrates multiple signals—such as hormones, sugar dynamics, and environmental signals—is needed to comprehensively decode the regulatory mechanisms of axillary bud development. This study advances our theoretical understanding of axillary bud regulation in plants, illuminates the interaction between developmental programs and environmental factors, and provides a conceptual foundation for molecular design breeding strategies.

## Data Availability

All original data supporting the findings of this study are available in the article/Supplementary Materials, and further inquiries can be directed to the corresponding author.

## Supplementary Materials

The following supporting information can be downloaded at: https://www.mdpi.com/article/10.3390/plants14243830/s1, Figure S1: DEG analysis related to hormones and environmental signals. Figure S2: Prediction Analysis of Transcription Factors in DEGs. Figure S3: The analysis of DEGs at the early response stage (Venn diagrams and GO enrichment). Figure S4: Phenotype of SL-related mutant induced by decapitation. Figure S5: Transcriptional expression of *NtEXB1* and *NtSTM* in various tissues. Figure S6: Function verification of *NtSTM*. Figure S7: Analysis of *NtEXB1* gene Information. Table S1: Summary table of transcriptome data.
