# Supplementary material for: Decapitation Rapidly Triggers Axillary Bud Release via Regulatory Network Reprogramming in Nicotiana tabacum
Source: Plants (Basel). 2025 Dec 16;14(24):3830. doi: 10.3390/plants14243830 (PMC12736617; doi:10.3390/plants14243830)
Supplement: Supplementary file 1 [file plants-14-03830-s001.zip › Supplementary Figures.pdf]

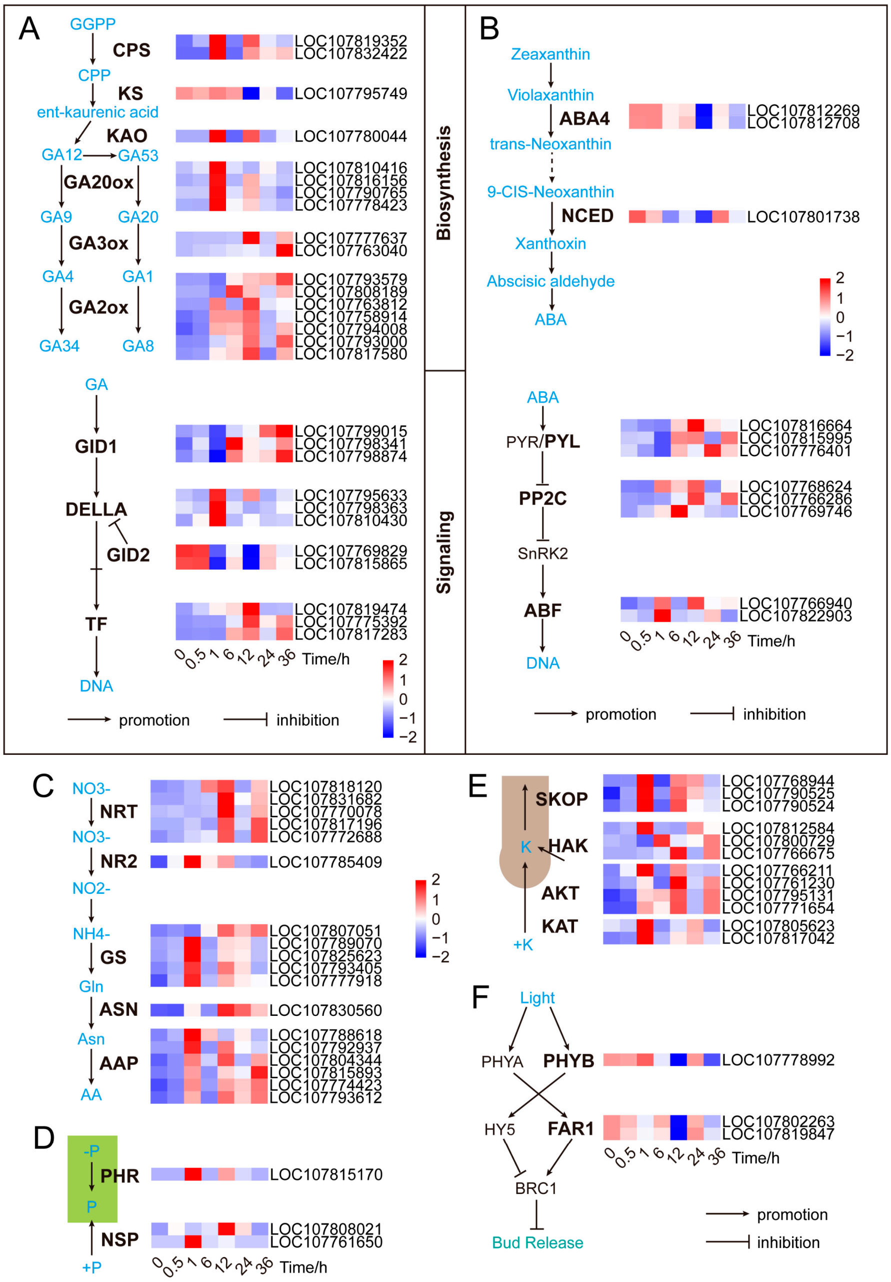

Figure S1. Analysis of hormone and environmental signaling-related DEGs. (A) Expression patterns of DEGs associated with gibberellin biosynthesis and signaling pathways at different time points after decapitation. (B) Expression patterns of DEGs related to abscisic acid biosynthesis and signaling pathways. (C) Expression profiles of key genes involved in nitrogen uptake and transport. (D) Expression patterns of key genes in the phosphorus signaling pathway. (E) Expression profiles of key genes related to potassium uptake and transport. (F) Expression patterns of key genes in the light signaling pathway. In all heatmaps, red and blue shades represent up-regulated and down-regulated genes, respectively. DEGs were identified using thresholds of $|\log_2 \text{(fold change)}| \geq 2$ and Q value ≤ 0.05.

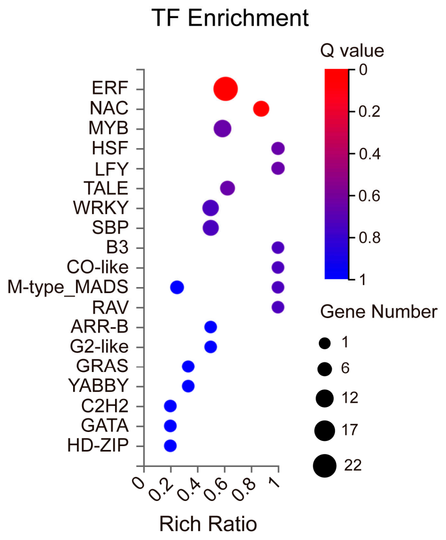

Figure S2. Predictive analysis of transcription factors among DEGs. The bubble size corresponds to gene count, while color intensity represents enrichment significance based on $-\log_{10}$ (Q value). Red and blue shades represent significantly and non-significantly enriched terms, respectively. DEGs were identified using thresholds of $|\log_2(\text{fold change})| \geq 2$ and Q value ≤ 0.05.

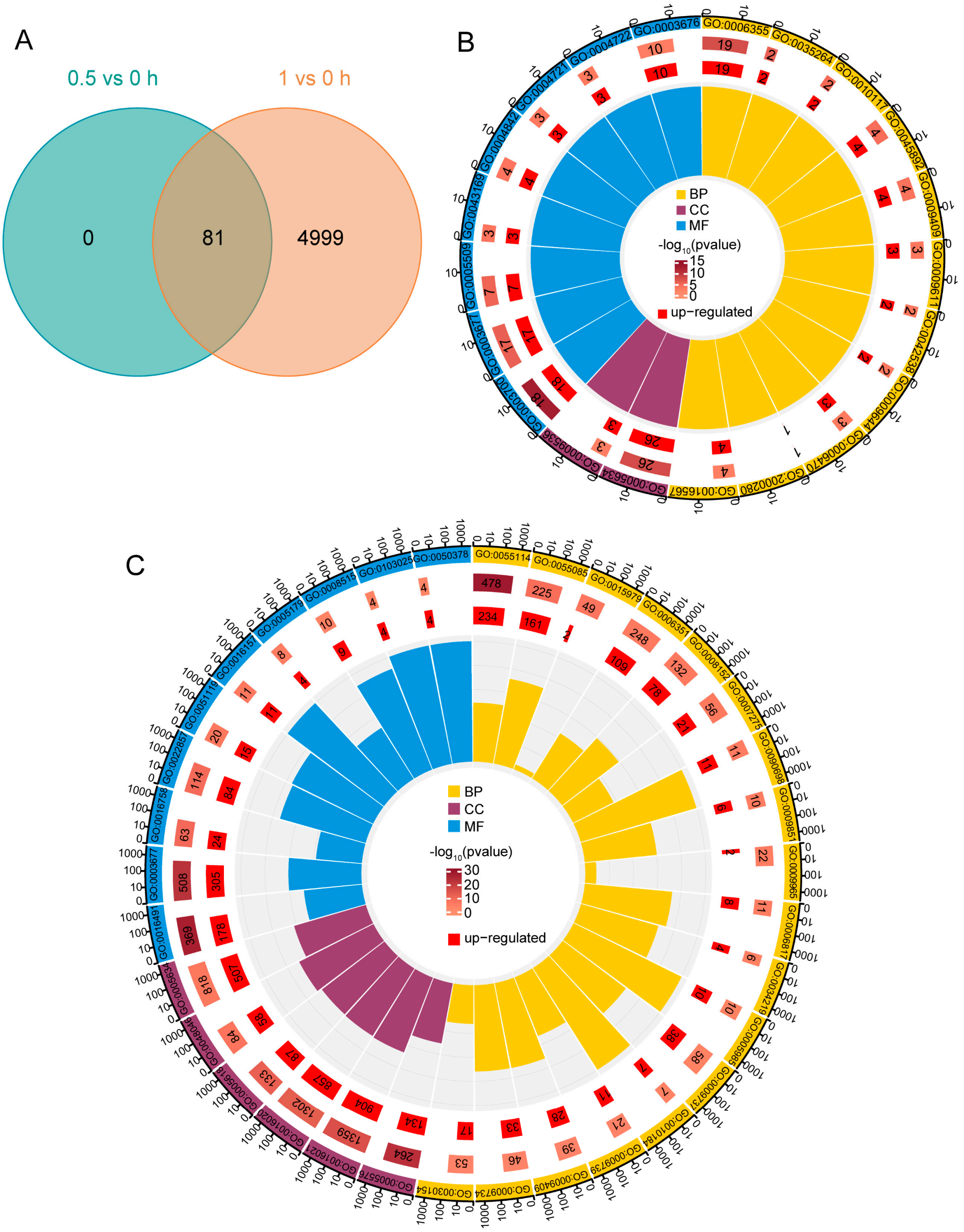

Figure S3. Analysis of DEGs at the very early stage after decapitation. (A) Venn diagram showing DEGs at the very early stage (0.5 and 1 hour) after decapitation. (B, C) GO enrichment analysis of DEGs at 0.5 h (B) and 1 h (C) after decapitation, respectively. BP, Biological Process; CC, Cellular Component; MF, Molecular Function. Red bars and associated numbers indicate the count of up-regulated genes in each term, while the color gradient represents the enrichment significance based on –log$_{10}$ (Q value).

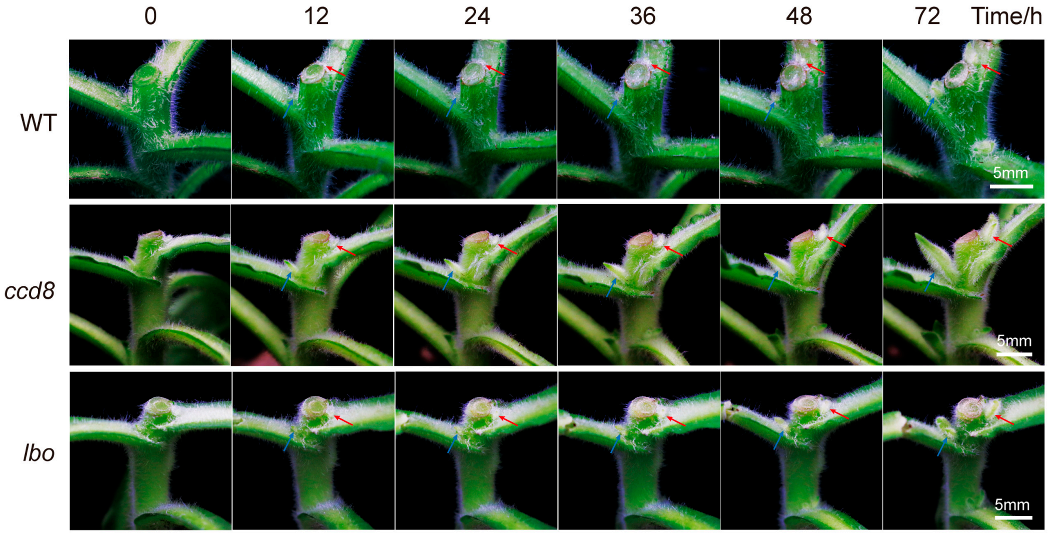

Figure S4. Phenotype of strigolactone mutants in response to decapitation. Axillary bud development was observed in eight-leaf-stage wild-type (WT) plants and strigolactone biosynthetic mutants (*ccd8*, *lbo*) at various time points (0, 12, 24, 36, 48, 72 hours) after decapitation. Red and blue arrows indicate axillary buds at the first and second leaf positions, respectively. Scale bar, 5 mm.

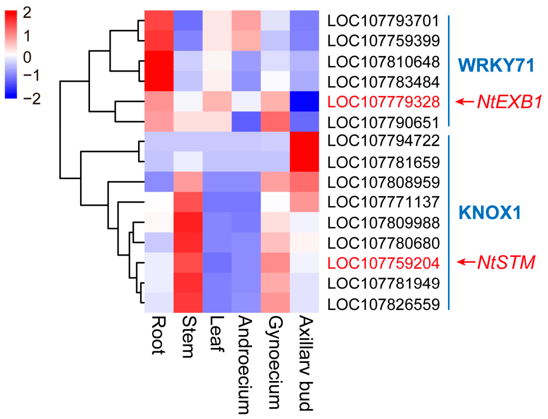

Figure S5. Transcriptional profiles of *NtEXB1* and *NtSTM* across various tissues. Genes highlighted in red indicate the selected target genes: *NtEXB1* (*LOC107779328*), a WRKY71 transcription factor family member; and *NtSTM* (*LOC107759204*), a KNOX1 transcription factor family member. Tissue abbreviations: Root; Stem; Leaf; Androecium; Gynoecium; Axillary bud. In the heatmap, red and blue shades represent relatively high and low expression levels, respectively, with normalized expression values shown for reference. DEGs were identified using thresholds of $|\log_2 (\text{fold change})| \geq 2$ and Q value ≤ 0.05.

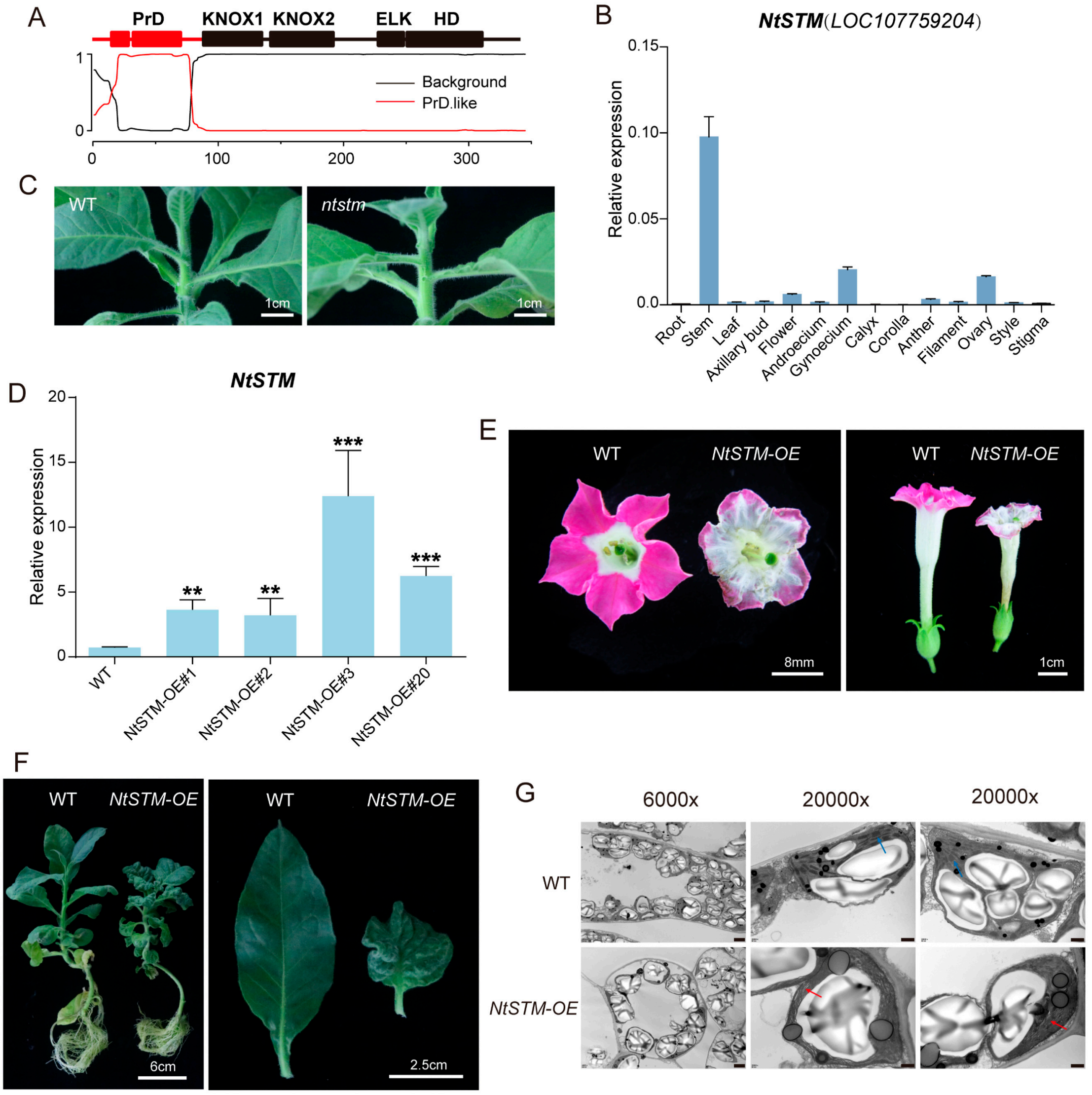

Figure S6. Functional validation of *NtSTM* in tobacco. (A) Conserved domain analysis of NtSTM, showing the typical "KNOX1" and "KNOX2" domains of the KNOX family. The red segment (PrD) indicates the intrinsic disordered region (IDR). (B) Tissue-specific expression profile of *NtSTM* in various tissues of flowering-stage plants. (C) Phenotype of the *ntstm* mutant compared to wild-type (WT) control at the seven-leaf stage. Scale bar, 1 cm. (D) Expression levels of *NtSTM* in different overexpression lines. (E) Flower phenotypes of *NtSTM*-overexpressing (OE) plants compared to WT. Scale bars, 8 mm and 1 cm. (F) Plant architecture and leaf phenotypes of *NtSTM-OE* plants versus WT. Scale bars, 6 cm and 2.5 cm. (G) Ultrastructural observation of leaves in *NtSTM-OE* plants. Blue arrows indicate normally aligned grana thylakoids; red arrows indicate disorganized grana lamellae. Scale bars: 2 μm at 6,000× magnification; 500 nm at 20,000× magnification. Error bars represent standard error (n = 3). Asterisks indicate statistically significant differences (*P < 0.05, significantly correlated; **P < 0.01 and ***P < 0.001, highly significantly correlated; Student's t-test).

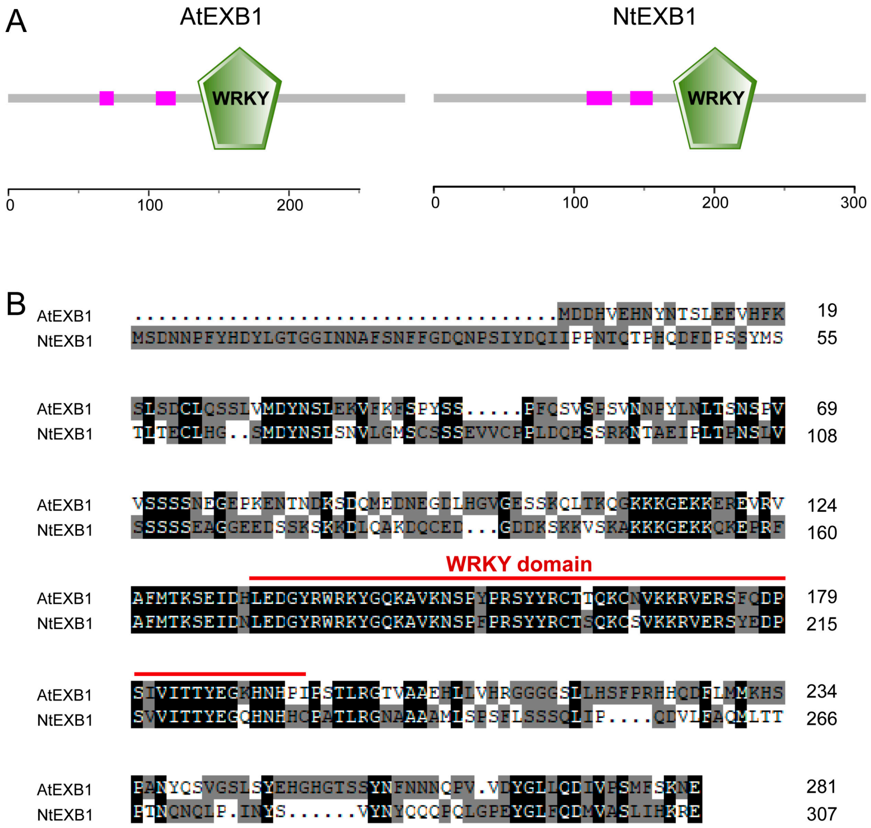

Figure S7. Analysis of *NtEXB1* gene information. (A) Conserved domain analysis of EXB1 proteins from *Arabidopsis thaliana* (At) and *Nicotiana tabacum* (Nt). (B) Amino acid sequence alignment of AtEXB1 and NtEXB1. The "WRKY" domain represents the characteristic functional domain of the WRKY transcription factor family.
